# Supplementary material for: A CRISPRi/a screening platform to study cellular nutrient transport in diverse microenvironments
Source: Nat Cell Biol. 2024 Apr 11;26(5):825–38. doi: 10.1038/s41556-024-01402-1 (PMC11098743; doi:10.1038/s41556-024-01402-1)
Supplement: Supplementary file 1 — Reporting Summary [file 41556_2024_1402_MOESM1_ESM.pdf]

nature portfolio

Corresponding author(s): Christopher Chidley; Peter Sorger

Last updated by author(s): Mar 1, 2024

Reporting Summary

Nature Portfolio wishes to improve the reproducibility of the work that we publish. This form provides structure for consistency and transparency in reporting. For further information on Nature Portfolio policies, see our Editorial Policies and the Editorial Policy Checklist.

Statistics

For all statistical analyses, confirm that the following items are present in the figure legend, table legend, main text, or Methods section.

n/a	Confirmed	
☐	☒	The exact sample size (*n*) for each experimental group/condition, given as a discrete number and unit of measurement
☐	☒	A statement on whether measurements were taken from distinct samples or whether the same sample was measured repeatedly
☐	☒	The statistical test(s) used AND whether they are one- or two-sided<br>*Only common tests should be described solely by name; describe more complex techniques in the Methods section.*
☒	☐	A description of all covariates tested
☐	☒	A description of any assumptions or corrections, such as tests of normality and adjustment for multiple comparisons
☐	☒	A full description of the statistical parameters including central tendency (e.g. means) or other basic estimates (e.g. regression coefficient) AND variation (e.g. standard deviation) or associated estimates of uncertainty (e.g. confidence intervals)
☐	☒	For null hypothesis testing, the test statistic (e.g. F, t, r) with confidence intervals, effect sizes, degrees of freedom and P value noted<br>*Give P values as exact values whenever suitable.*
☒	☐	For Bayesian analysis, information on the choice of priors and Markov chain Monte Carlo settings
☒	☐	For hierarchical and complex designs, identification of the appropriate level for tests and full reporting of outcomes
☐	☒	Estimates of effect sizes (e.g. Cohen's *d*, Pearson's *r*), indicating how they were calculated

Our web collection on statistics for biologists contains articles on many of the points above.

Software and code

Policy information about availability of computer code

Data collection	Western blots were imaged on an ImageQuant LAS4000 (Cytiva) or an iBright FL1500 Imaging System (Invitrogen).<br>Luminescence from Cell-Titer Glo assays was measured on a plate reader (BioTek Synergy H1).<br>qPCR results were collected on a QuantStudio 6 Real-Time PCR system (Thermo Fisher Scientific).<br>The composition of pooled libraries was determined by sequencing using an Illumina HiSeq 2500 platform and a 50 bp single read on a high output standard v4 flow cell, or using an Illumina NextSeq 500 platform and a 75 bp single read on a high output flow cell.<br>Flow cytometry was collected on a BD LSRFortessa or a BD Biosciences LSR II.<br>Derivatized amino acid samples were analyzed on an Agilent 7890B gas chromatograph linked to an Agilent 5977B mass spectrometer.<br>No other specific software or code was used for data collection.
Data analysis	CRISPR screens were analyzed using an open source code available at https://github.com/mhorlbeck/ScreenProcessing.<br>All other data was analyzed using R (v. 3.6.0). General Data visualization was performed using ggplot2 (v. 3.3.3). Chord plots were generated with GOplot (v. 1.0.2). Pairwise Pearson correlations (and P values) were calculated using the function rcorr in the R package Hmisc (v. 4.7.1) and data was displayed using the R package corrplot (v. 0.84).<br>GC-MS raw data was quantified using El-MAVEN (v. 0.11.0) and natural isotope abundances were corrected using IsoCorrectoR (v. 1.2.1).

For manuscripts utilizing custom algorithms or software that are central to the research but not yet described in published literature, software must be made available to editors and reviewers. We strongly encourage code deposition in a community repository (e.g. GitHub). See the Nature Portfolio guidelines for submitting code & software for further information.

Data

Policy information about availability of data

All manuscripts must include a data availability statement. This statement should provide the following information, where applicable:

- Accession codes, unique identifiers, or web links for publicly available datasets
- A description of any restrictions on data availability
- For clinical datasets or third party data, please ensure that the statement adheres to our policy

All CRISPR screening data are provided in Supplementary Tables 5,6. The composition of the sgRNA libraries is provided in Supplementary Table 1. Source data have been provided in Source Data files. All other data supporting the findings of this study are available from the corresponding author on reasonable request.

Research involving human participants, their data, or biological material

Policy information about studies with human participants or human data. See also policy information about sex, gender (identity/presentation), and sexual orientation and race, ethnicity and racism.

Reporting on sex and gender	not applicable
Reporting on race, ethnicity, or other socially relevant groupings	not applicable
Population characteristics	not applicable
Recruitment	not applicable
Ethics oversight	not applicable

Note that full information on the approval of the study protocol must also be provided in the manuscript.

Field-specific reporting

Please select the one below that is the best fit for your research. If you are not sure, read the appropriate sections before making your selection.

☒ Life sciences ☐ Behavioural & social sciences ☐ Ecological, evolutionary & environmental sciences

For a reference copy of the document with all sections, see nature.com/documents/nr-reporting-summary-flat.pdf

Life sciences study design

All studies must disclose on these points even when the disclosure is negative.

Sample size	We performed experiments to determine the implantation rate of K562 leukemia cells in the subcutaneous flank tissue of NSG mice. Our initial experiments determined that sufficient library representation was achieved in each single tumor. We decided to have 6 replicate tumors for each screen.<br>No sample size calculation was performed for experiments in cell culture. The sample size for each experiment is included in the respective figure legend. We included a minimum of 3 biologically independent experiments.
Data exclusions	To improve data quality in the in vivo CRISPR screens, we excluded in an unbiased way 2 datasets from the 6 replicates that were collected for each condition. We excluded the 2 datasets with the highest amount of technical noise, which was determined by the lowest number of NTC sgRNA counts that were within 1 log2 of the median after growth in vivo.
Replication	Transporter CRISPR screens were run in duplicates to quantify reproducibility. All attempts at replication of transporter CRISPR screens were successful, as shown in Extended Data Fig 2c,d.<br>All screens include 10 sgRNAs per gene and non targeting controls to evaluate biological noise. The number of independent replicates for all other experiments is mentioned in the corresponding figure legend.
Randomization	Mice were randomly chosen for the 4 subcutaneous injection conditions. All pooled CRISPR screens are inherently randomized. Other experiments were not randomized as there was no allocation into different experimental groups.
Blinding	The researcher performing subcutaneous injections was blinded to the identity of the cells injected. All pooled CRISPR screens are inherently blinded. Other experiments were not blinded as all data was collected using unbiased methods.

Reporting for specific materials, systems and methods

We require information from authors about some types of materials, experimental systems and methods used in many studies. Here, indicate whether each material, system or method listed is relevant to your study. If you are not sure if a list item applies to your research, read the appropriate section before selecting a response.

Materials & experimental systems

n/a	Involved in the study
☐	☒ Antibodies
☐	☒ Eukaryotic cell lines
☒	☐ Palaeontology and archaeology
☐	☒ Animals and other organisms
☒	☐ Clinical data
☒	☐ Dual use research of concern
☒	☐ Plants

Methods

n/a	Involved in the study
☒	☐ ChIP-seq
☐	☒ Flow cytometry
☒	☐ MRI-based neuroimaging

Antibodies

Antibodies used

Cell Signaling Technology primary rabbit antibodies: vinculin (E1E9V) #13901 Dilution 1:2000; Phospho-p70 S6 Kinase (Thr389) #9234 Dilution 1:1000; p70 S6 Kinase #9202 Dilution 1:1000; Phospho-eIF2α (Ser51) (D9G8) #3398 Dilution 1:1000; eIF2α #9722 Dilution 1:2000; Phospho-4E-BP1 (Ser65) #9451 Dilution 1:1000; 4E-BP1 (53H11) #9644 Dilution 1:2000; SLC3A2/4F2hc (D603P) #13180 Dilution 1:1000; SLC2A1 (D3J3A) #12939 Dilution 1:1000.
Other primary antibodies: SLC7A5 (Proteintech, 28670-1-AP) Dilution 1:5000; SLC7A6 (Novus Biologicals, NBP2-75086) Dilution 1:500; SLC7A7 (Novus Biologicals, NBP1-82826) Dilution 1:500.
Secondary antibodies: anti-rabbit secondary antibody (IRDye800CW LI-COR 926-32211) Dilution 1:20,000; anti-goat secondary antibody (Donkey IgG H&L, Alexa Fluor 750 conjugate, Abcam ab175744) Dilution 1:10,000; goat HRP-linked Anti rabbit IgG (Cell Signaling Technology #7074) Dilution 1:5000.

Validation

Cell Signaling Technology primary antibodies used in this study are well-published commercial antibodies. Validation statements for use of these antibodies in Western blots and literature references are available on the manufacturer's website (https://www.cellsignal.com/).
Other primary antibodies have validation statements on the supplier's website for use in Western blots (https://www.ptglab.com/ and https://www.novusbio.com/).
The specificity for antibodies against SLC2A1, SLC7A5, SLC7A6, SLC7A7 was confirmed in this study using CRISPRi/a cell lines.

Eukaryotic cell lines

Policy information about cell lines and Sex and Gender in Research

Cell line source(s)	Cell lines were from ATCC: K562 (CCL–243), A375 (CRL–1619), C2BBe1 clone of Caco-2 (CRL–2102), HEK293T (CRL–3216).
Authentication	Cells lines were used from low passage from ATCC stocks. Cell lines were not further authenticated.
Mycoplasma contamination	All cell lines were confirmed mycoplasma free using the MycoAlert mycoplasma detection kit (Lonza LT07-318).
Commonly misidentified lines (See ICLAC register)	none

Animals and other research organisms

Policy information about studies involving animals; ARRIVE guidelines recommended for reporting animal research, and Sex and Gender in Research

Laboratory animals	NOD.Cg-Prkdcscid Il2rgtm1Wjl/SzJ mice (NSG mice; The Jackson Laboratory, Strain #005557). Male mice between 3 and 4 months old were used in this study.
Wild animals	Study did not involve wild animals.
Reporting on sex	Male mice were used in this study
Field-collected samples	Study did not involve Field-collected samples
Ethics oversight	All animal experiments conducted in this study were approved by the Massachusetts Institute of Technology (MIT) Institutional Animal Care and Use Committee (IACUC).

Note that full information on the approval of the study protocol must also be provided in the manuscript.

Flow Cytometry

Plots

Confirm that:

☒ The axis labels state the marker and fluorochrome used (e.g. CD4-FITC).

☒ The axis scales are clearly visible. Include numbers along axes only for bottom left plot of group (a 'group' is an analysis of identical markers).

☒ All plots are contour plots with outliers or pseudocolor plots.

☒ A numerical value for number of cells or percentage (with statistics) is provided.

Methodology

Sample preparation	K562 cells in culture in RPMI. Washed once with PBS and strained through 35 micron mesh filter
Instrument	BD FACS Aria II
Software	BD FACSDiva 8.0.1
Cell population abundance	BFP+ cells represented about 30-50% of initial population. We performed 2 rounds of cell sorting, isolating the top half of BFP + cells and then single cell cloned. The resulting line is 100% pure
Gating strategy	Untransduced K562 cells were used to define BFP- cells. We determined the BFP+ cells in transduced K562 and gated on the top half of this population.

☒ Tick this box to confirm that a figure exemplifying the gating strategy is provided in the Supplementary Information.
