# Supplementary material for: A CRISPRi/a screening platform to study cellular nutrient transport in diverse microenvironments
Source: Nat Cell Biol. 2024 Apr 11;26(5):825–38. doi: 10.1038/s41556-024-01402-1 (PMC11098743; doi:10.1038/s41556-024-01402-1)

Western blots Raw Data #1 (Extended Data Figure 1c)

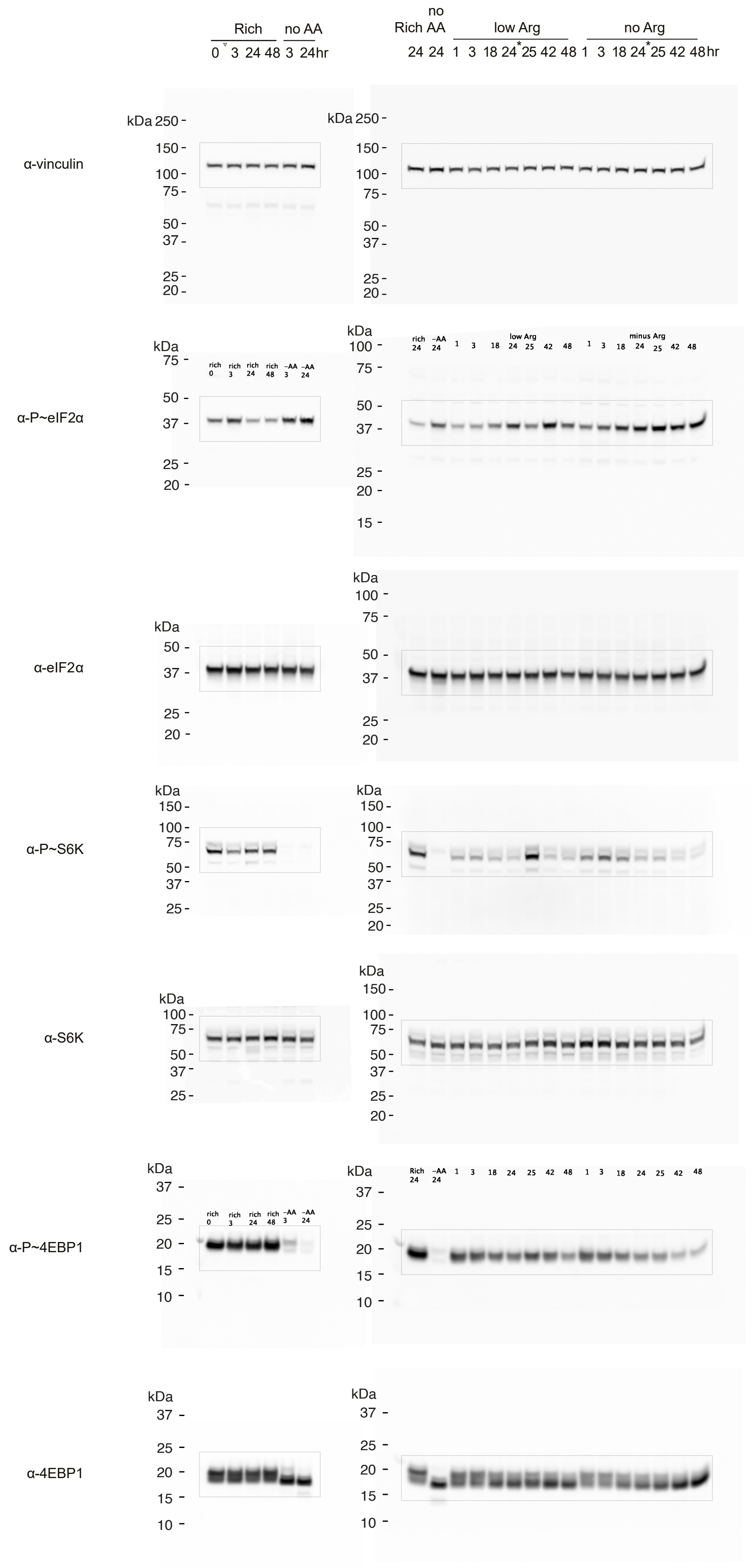

Western blots Raw Data #2 (Extended Data Figure 1c)

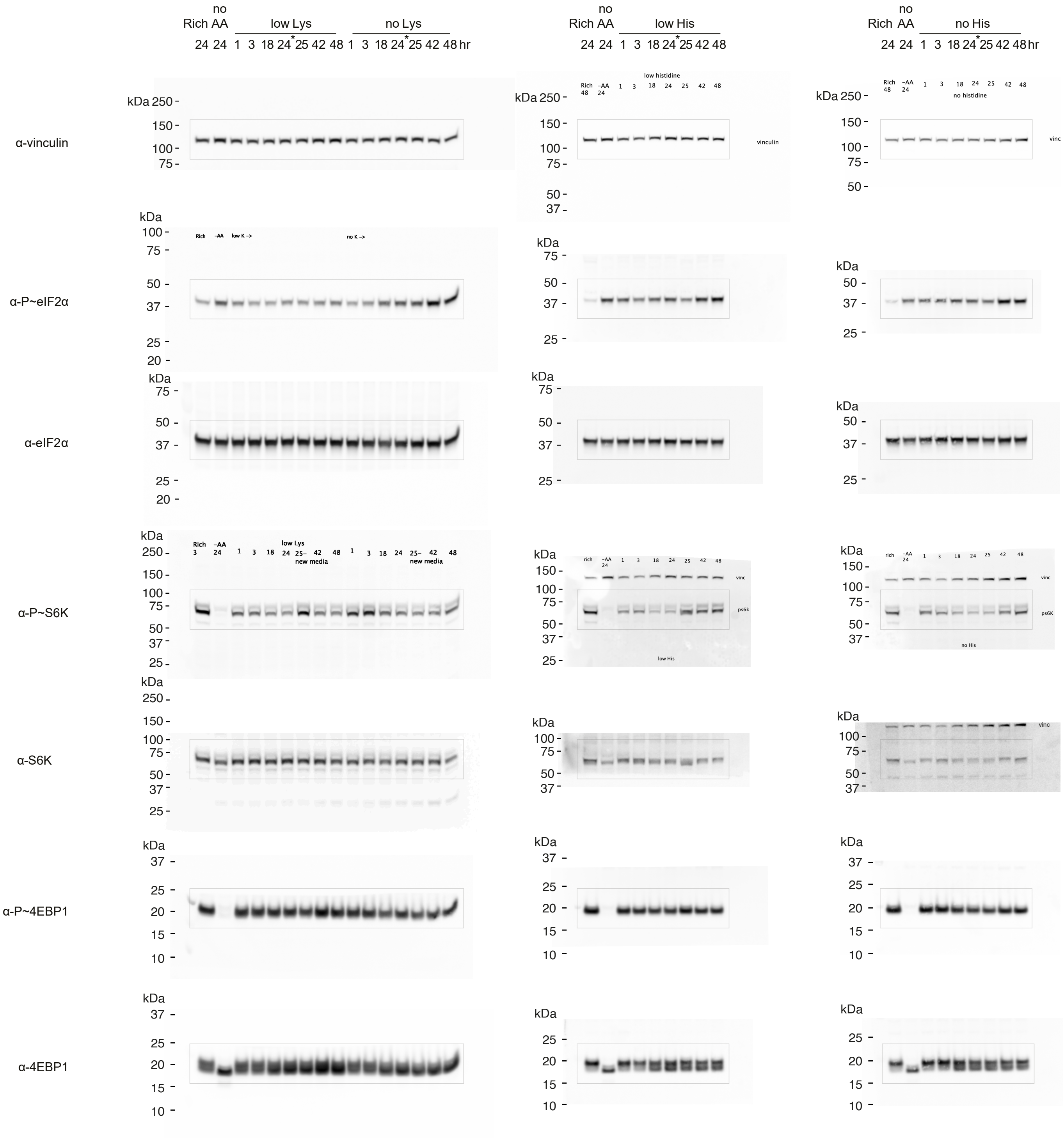

Western blots Raw Data #3 (Figure 1c and Extended Data Figure 2a)

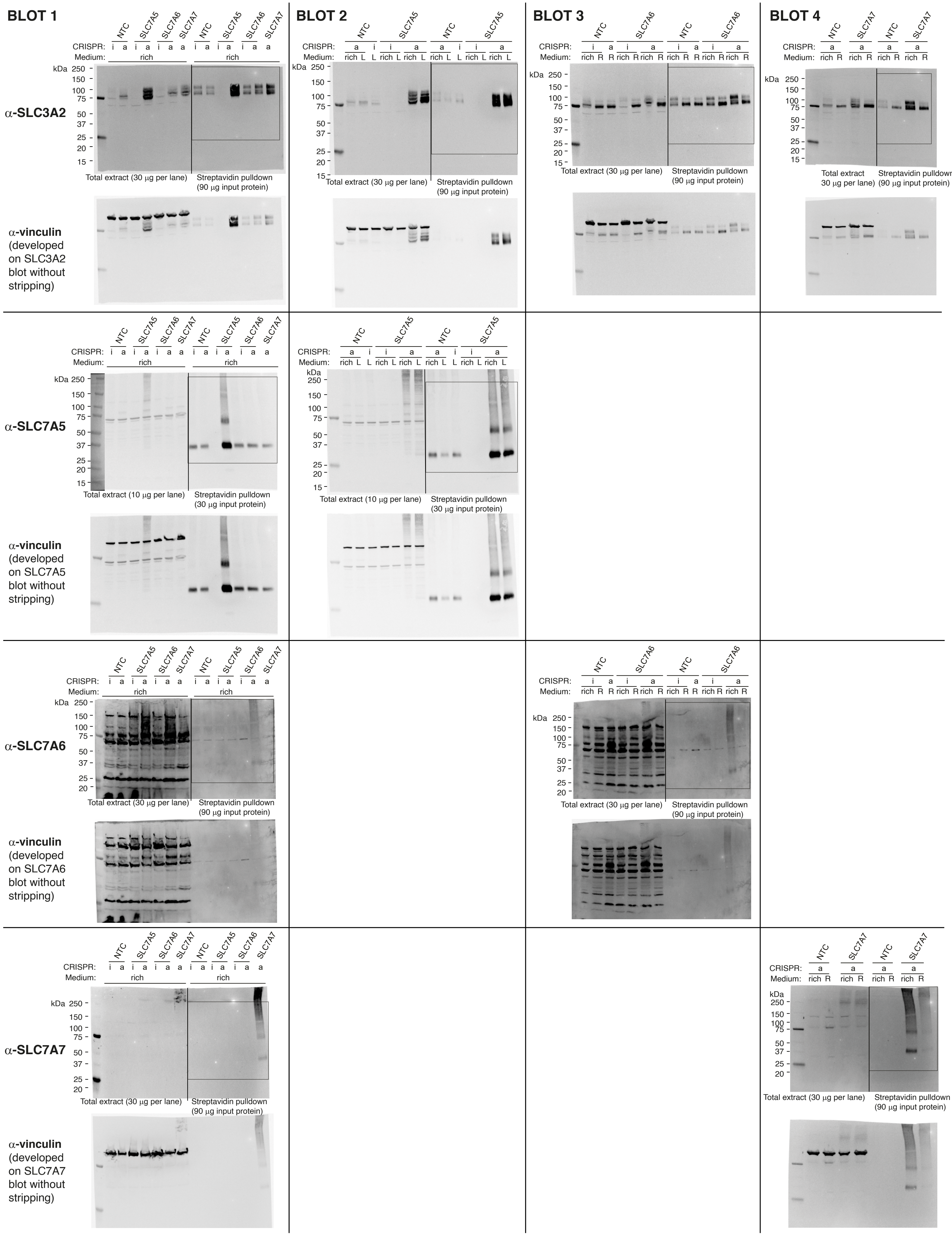

Western blots Raw Data #4 (Extended Data Figure 8d)

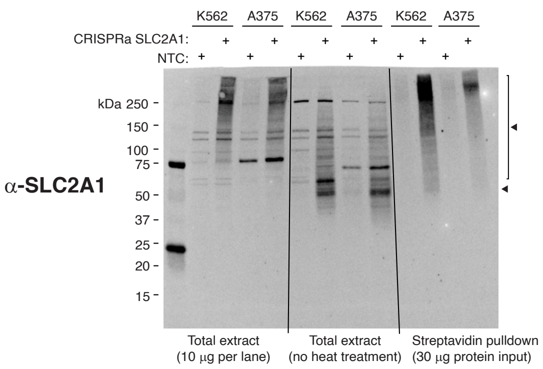

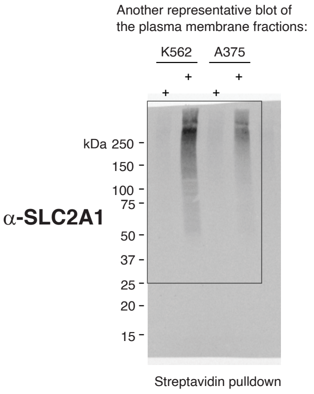

α-SLC2A1

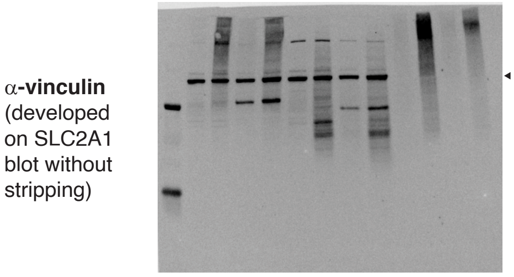

Supplement: Supplementary file 23 — Unprocessed western blots. One file for all blots. [file 41556_2024_1402_MOESM23_ESM.pdf]
